# Primary bilateral macronodular adrenocortical hyperplasia (PBMAH) patient with *ARMC5* mutations

**DOI:** 10.1186/s12902-023-01324-3

**Published:** 2023-04-07

**Authors:** Peng Tang, Jun Zhang, Song Peng, Xuzhi Yan, Yapeng Wang, Shuo Wang, Yao Zhang, Gaolei Liu, Jing Xu, Yiqiang Huang, Dianzheng Zhang, Qiuli Liu, Jun Jiang, Weihua Lan

**Affiliations:** 1 Department of Urology, Daping Hospital, Army Medical University, 10#, ChangjiangZhilu, Yuzhong District, Chongqing, 400042 People’s Republic of China; 2 Department of Bio-Medical Sciences, Philadelphia College of Osteopathic Medicine, 4170 City Avenue, Philadelphia, PA 19131 USA

**Keywords:** Primary bilateral macronodular adrenocortical hyperplasia, *ARMC5*, Sequencing, Adrenal venous sampling, Cushing syndrome

## Abstract

**Background:**

Primary bilateral macronodular adrenocortical hyperplasia (PBMAH) is a highly heterogeneous disease with divergent manifestations ranging from asymptomatic subclinical Cushing syndrome (CS) to overt Cushing syndrome with severe complications. *ARMC5* mutations occur in 20 to 55% PBMAH patients usually with more severe phenotypes. Different *ARMC5* mutations might be associated with diverse phenotypes of PBMAH.

**Case presentation:**

A 39-year-old man was admitted to our hospital with progressive weight gain and severe hypertension. He presented typical CS and its classical metabolic and bone complications like hypertension and osteoporosis. The laboratory results showed high levels of cortisol and low levels of ACTH. Low- and high-dosed dexamethasone suppression tests were negative. Contrast-enhanced computed tomography (CT) revealed multiple bilateral irregular macronodular adrenal masses. Adrenal venous sampling (AVS) confirmed that the right adrenal gland with larger nodules secreted more hormone that the left side did. Right adrenalectomy and subsequent contralateral subtotal resection were conducted. His blood pressure and CS symptoms as well as comorbidities including backache and muscle weakness improved. Whole exome sequencing identified one *ARMC5* germline mutation (c.1855C > T, p. R619*), five *ARMC5* somatic mutations (four novel mutations) in his right and left adrenal nodules.

**Conclusions:**

This PBMAH patient was identified with one *ARMC5* germline mutation and five different somatic *ARMC5* mutations (four novel mutations) in the different nodules of the bilateral adrenal masses. AVS combined with CT imagine could be helpful to determine the dominant side for adrenalectomy. Genetic testing is important for the diagnosis and management of the patient with PBMAH.

**Supplementary Information:**

The online version contains supplementary material available at 10.1186/s12902-023-01324-3.

## Background

Primary bilateral macronodular adrenocortical hyperplasia (PBMAH) is a highly heterogeneous disease and variable levels of cortisol produced by bilateral benign adrenocortical macronodules larger than 10 mm [1]. Patients with PBMAH present with divergent phenotypes ranging from asymptomatic subclinical Cushing syndrome (SCS) to overt Cushing syndrome (CS) with severe complications. Overt CS includes weight gain, proximal myopathy, buffalo neck, red stretch marks, skin fragility, easy bruising, hyperandrogenism, and classical metabolic and bone complications such as hypertension, diabetes, venous thrombo-embolism, osteopenia, and osteoporosis [2]. Most patients with clinical symptoms are diagnosed between 40 and 60 years of age and the diagnosis is often made only after several years or decades of disease progression indicating that the tumor growth and cortisol dysregulation progress slowly and silently [1]. Mild autonomous cortisol secretion is associated with absent or moderate symptoms, thus most PBMAH patients are identified incidentally during computed tomography and magnetic resonance imaging for unrelated conditions [2], which makes early diagnosis challenging.

The bilateral nature of the adrenal lesions and the description of familial cases of patients with PBMAH provide support for the hypothesis of a germline genetic predisposition [2]. Previous studies have shown that inactivating germline mutations of tumor suppressor genes including *MEN1*, *APC* and *FH* could result in PBMAH accompanied by other syndromic presentations such as primary hyperparathyroidism, neuroendocrine tumors, pituitary adenomas (MEN1 syndrome, multiple endocrine neoplasia type 1), colon polyps (FAP, familial adenomatous polyposis), leiomyomas, leiomyosarcomas and renal cancer (HLRCC, hereditary leiomyomatosis and renal cell cancer) [3]. *ARMC5* pathogenic mutations occur in 20 to 55% of patients with PBMAH [1, 4, 5]. Noteworthy, patients carrying *ARMC5* mutations usually have earlier onset, bigger nodules, higher cortisol levels, and more severe symptoms [4]. It has been noticed that some symptoms appear to be *ARMC5* mutation-specific [6]. PBMAH patients with *ARMC5* mutations are more often treated surgically [2]. From this point of view, identifying PBMAH patients with *ARMC5* mutations might be important for the treatment decision-making. Here we report a PBMAH patient carrying one germline mutation and five somatic mutations in *ARMC5*.

## Case presentation

### Clinical features

**Fig. 1:**
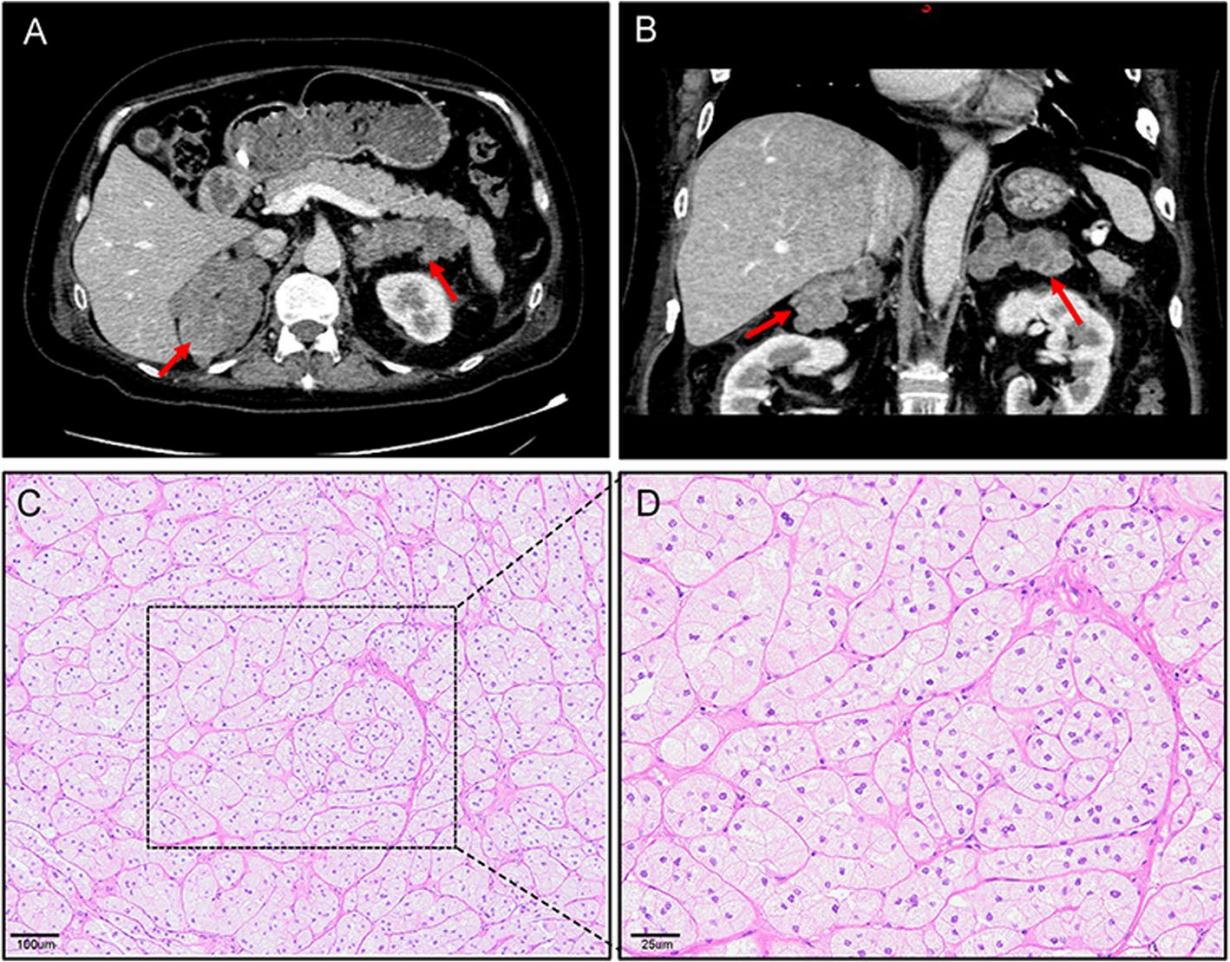
Results of imaging and histopathology. **A** and **B** Adrenal computed tomography scan revealed bilateral adrenal masses (right: 7.2 × 5.2 × 5.1 cm, left: 6.9 × 4.5 × 3.2 cm, indicated by red arrows). **C** (X100, OLYMPUS microscope, OLYMPUS BX53) and **D** (X400, OLYMPUS microscope, OLYMPUS BX53) Hematoxylin and eosin staining of the adrenal tissue revealed nodular hyperplasia

A 39-year-old male was admitted to our hospital with progressive weight gain for 8 years from 60 kg (BMI: 24.7 kg/m^2^) to 71.8 kg (BMI: 29.4 kg/m^2^) and severe hypertension refractory to antihypertensive drugs for 2 years (150–180/100–120 mmHg). Physical examination showed multiple clinical manifestations of CS such as moon face, centripetal obesity, abdominal purple striae, limb edema, and muscle weakness. The hormonal work-up on admission confirmed significantly increased serum cortisol and urinary free cortisol (UFC) as shown in Table 1. In addition, his plasma ACTH levels were suppressed markedly at all time points, especially at 16:00 (2.69 pg/mL, normal ranges: 10.7–30.5 pg/mL). The results of LD-DST and HD-DST were both negative. All these findings suggested that his hypercortisolism was ACTH-independent. In addition, he had low level of testosterone (0.1 ng/ml, normal range: 1.75–7.81 ng/mL), which could happen due to decreased gonadal function in severe Cushing syndrome, while the levels of LH (1.62mIU/mL, 1.24–8.62mIU/mL) and FSH (4.21mIU/mL, normal range: 1-8mIU/mL) are normal. Contrast-enhanced CT showed bilateral adrenal multiple irregular macronodular masses (right adrenal: 7.2 × 5.2 × 5.1 cm, left adrenal: 6.9 × 4.5 × 3.2 cm) (Fig. 1). Positron emission tomography/computed tomography (PET/CT) revealed vertebral (T4-T6) compression, osteoporosis, enlarged heart, as well as multiple nodules in the bilateral adrenal masses. The lower bone mineral density (BMD) further substantiated the osteoporosis. Adrenal venous sampling (AVS) was conducted to evaluate the predominant side of cortisol production (Table 2). Selectivity indexes (SI) were 1.77 and 3.04 in the left and right adrenals, respectively, indicating the success of AVS cannulation (SI cutoff of 1.4) [7]. However, there was no lateralization according to the lateralization index (LI < 4). Based on the absolute serum cortisol level, the right adrenal with larger nodules was confirmed to produce more cortisol than the left. Considering the AVS result and the sizes of the adrenal masses, adrenalectomy was conducted on his right adrenal gland and pathological examination revealed adrenal nodular hyperplasia (Fig. 1). One week after surgery, his serum and urinary free cortisol levels reduced significantly but remained at high levels. During the follow-up, the CS symptoms did not improve. Six months later, the patient was hospitalized and underwent subtotal left adrenalectomy. His blood pressure, CS symptoms and comorbidities including backache and muscle weakness improved thereafter.

**Table 1:** The main laboratory and hormone expression profile

Laboratory test (reference range)	On first admission	One week after right adrenalectomy	Before left adrenalectomy
Cortisol (8 a.m.), ng/mL (50–280 ng/mL)	417	269	234
Cortisol (4 p.m.), ng/mL (20–140 ng/mL)	320	249	213
Cortisol (12 a.m.), ng/mL (10–120 ng/mL)	329	119	169
ACTH (8 a.m.), pg/mL (5.08–32.8 pg/mL)	5.43	< 1.6	< 1.6
ACTH (4 p.m.), pg/mL (10.7–30.5 pg/mL)	2.69	< 1.6	< 1.6
ACTH (12 a.m.), pg/mL (5–15 pg/mL)	5.70	< 1.6	< 1.6
24-hour UFC, nmol/24H, 160–1112 nmol/24H	6318	5397	2631
Orthostatic renin activity, ng/mL/h (0.33–5.15 ng/mL/h)	1.49	–	–
Clinostatic renin activity, ng/mL/h (0.07–1.51 ng/mL/h)	0.62	–	–
WBC, (4–10 × 10^9^/L)	13.8	9.22	11.63
Hemaglobulin	158	123	163
Platelet, (100–300 × 10^9^/L))	219	192	247
eGFR, ml/min/1.73 m^2^	171.42	307.11	
Creatinine	60	36.2	49.1
K+	2.24–3.42	2.59–3.58	3.28
BNP	702.97	368.32	–

*ACTH* adrenocorticotropic hormone, *UFC* urinary-free cortisol, *ARR* aldosterone renin ratio, *WBC* white blood cell, *eGFR* estimated glomerular filtration rate, *BNP* brain natriuretic peptide

**Table 2:** Biochemical results from AVS

	Inferior vena cava	Left adrenal	Right adrenal	SI Left	SI Right	LI L/R ratio	LI R/L ratio
Cortisol (nmol/L)	941	1664	2869	1.77	3.04	2.71	0.37
Aldosterone (ng/dL)	2.83	3.73	17.4	1.32	6.15		

SI = (cortisol or aldosterone) _right or left darnel_/(cortisol or aldosterone)_inferior vena cava_; LI = (cortisol/ aldosterone) _one side_/ (cortisol/ aldosterone) _another side_

### Identification of the mutations

**Fig. 2:**
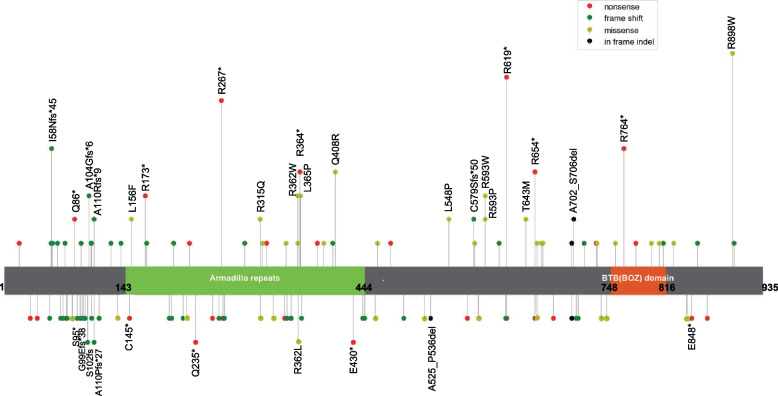
Detailed mutation landscape of *ARMC5*. The upper of the box represents the germline mutations and lower represents the somatic mutations. The identified mutations in this study and recurrent mutations previously reported are named with their amino acid alterations

Whole exome sequencing (WES) was conducted on peripheral blood leukocytes and bilateral adrenal tumor tissues. A heterozygous germline mutation in *ARMC5* (c. 1855C > T, p. R619*) was identified with 23 and 27 somatic mutations in his right and left adrenal, respectively (Table 3, Supplementary Table 1 and supplementary Table 2). Of note, three *ARMC5* somatic mutations were detected in the right adrenal lesion, and two *ARMC5* somatic mutations were found in the left adrenal gland (Table 3). Among them, four somatic mutations (p.A525_P536del, p.G99Efs*38, p.S95*, p.E848*) have not been previously reported in public databases and were predicted probably damaging by in silico models (MutationTaster and SIFT). One of them were long InDel and another three were substitutions leading to nonsense mutations. These novel mutations contributed to the mutation landscape of *ARMC5* (Fig. 2) [8]. None of his family members had any history of endocrine disease and all of them refused to have their genes tested.

**Table 3:** The somatic *ARMC5* variants in the bilateral adrenal mass

Location	Gene symbol	Transcripts	Genomic location	cHGVS	pHGVS	ExIn ID	Mutation frequency	MutationTaster	SIFT	PolyPhen-2	ExAC or 1000G
Right adrenal	*ARMC5*	NM_001105247.1	16p11.2	c.284C > A	p.S95^*^	EX1	20.1%	Disease causing	Deleterious	Benign	−/−
Right adrenal	*ARMC5*	NM_001105247.1	16p11.2	c.294Gdel	p.G99Efs^*^38	EX1	10.4%	Disease causing	Deleterious	Benign	−/−
Right adrenal	*ARMC5*	NM_001105247.1	16p11.2	c.1572_1607delAGCCCTGCTGCTGCTGTCGCGCTTTTCCCAGGCCCC	p.A525_P536del	EX4	2.0%	Disease causing	Deleterious	Benign	−/−
Left adrenal	*ARMC5*	NM_001105247.1	16p11.2	c.435C > A ^a^	p.C145^*^	EX1	4.5%	Disease causing	Deleterious	Benign	−/−
Left adrenal	*ARMC5*	NM_001105247.1	16p11.2	c.2542G > T	p.E848^*^	EX6	2.1%	Disease causing	Deleterious	Benign	−/−

*HGVS* Human Genome Variation Society, *ExIn* exon-intron, *ExAC* the Exome Aggregation Consortium, *1000G* 1000 Genomes

^a^ this mutation has been reported in the Catalogue Of Somatic Mutations In Cancer (COSMIC)

## Discussion and conclusions

PBMAH is known as an autosomal positive disease characterized with multiple nodules in bilateral adrenal like “a bunch of grapes appearance” [9]. We report here a PBMAH patient presented with severe CS symptoms including moon face, centripetal obesity, purple striae of abdomen, limb edema, and muscle weakness, as well as its classical metabolic and bone complications like hypertension and osteoporosis. This patient carries one germline and 5 somatic pathogenic mutations in *ARMC5*. Of note, four of these somatic *ARMC5* mutations have not been identified previously. AVS confirmed that the larger mass in his right adrenal produced more cortisol. One side of total followed by contralateral subtotal adrenalectomy appeared to work well for this particular patient.

The diagnosis of PBMAH in our case mainly depend on following four criteria [10]: (1) typical clinical manifestations and Cushing syndromes: central obesity, hypertension, purple striae, osteoporosis, and etc.; (2) abnormal laboratory tests, such as increased plasma cortisol levels and UFC, cortisol rhythm disorder, ACTH inhibition; (3) adrenal CT: multiple nodules in bilateral adrenal cortex (larger than 10 mm); and (4) pathology shows adrenal nodular hyperplasia. PBMAH has been considered as “ACTH-independent macronodular adrenal hyperplasia (AIMAH)” until Louiset et al. showed that local ACTH paracrine production by steroidogenic cells could lead to cortisol excess [11]. Previous studies indicate that plasma ACTH is suppressed in PBMAH patients, which could be used to discriminate PBMAH from ACTH-dependent CS, such as Cushing disease, ectopic secretion of ACTH [12, 13]. However, PBMAH needs to be discriminated from other ACTH-independent diseases, especially primary pigmented nodular adrenal disease (PPNAD) which could also present with CS and bilateral adrenal lesions. Imaging characteristics of PPNAD are bilateral adrenal micronodules with normal adrenal weight and size [3]; and pathological result often reveals pigmented nodules in PPNAD [14]. About 88% PPNAD are the endocrine manifestation of Carney complex (CNC) characterized by multiple endocrine and nonendocrine neoplasms and spotty skin pigmentation [15].

PBMAH is a highly heterogeneous disease with various degrees of cortisol secretion and CS. Most of PBMAH patients are not identified until overt CS occurs, patients with subclinical CS are hardly noticed unless the small enlargement of the adrenal is incidentally discovered [16]. Therefore, the phenotypes of PBMAH patients are diverse. Previous studies have identified the genotype-phenotype correlation in PBMAH patients [16]. Patients with mutant *ARMC5* were diagnosed earlier with PBMAH with higher prevalence of overt CS and hypertension [16]. This patient carrying *ARMC5* mutations presented with severe CS and classical metabolic and bone complications such as hypertension and osteoporosis also support the notion that PBMAH patients with *ARMC5* mutations could have severe phenotypes. Previous studies have shown that the sizes of the adrenal masses are associated with the levels of cortisol and severity of Cushing syndrome [17, 18]. The large enough adrenal volume is needed to cause hypercortisolism, evidenced by that Cushing syndrome is usually observed in PBMAH patients with large adrenal masses [19]. Furthermore, ages of the PBMAH patients with *ARMC5* mutations are correlated with cortisol hypersecretion [20].

*ARMC5* is located at 16p11.2 with six exons and more than 80 mutations have been reported but without any identified hot spot [2]. *ARMC5* mutations occur in up to 55% of operated PBMAH patients [16, 21]. In this report, we find our PBMAH patient carries a heterozygous germline mutation in *ARMC5* (c.1855C > T) resulting in a premature stop codon (p. R619*) which has been previously reported as a frequently mutation in Japanese patients with PBMAH [1, 20]. Based on Knudson’s ‘two-hit’ theory of a tumor suppressor, a second somatic *ARMC5* mutation would be needed in the background of the germline mutation [1]. Indeed, we found three and two (4 of them were previously unreported) side-specific *ARMC5* somatic mutations in his right and left adrenal tumors, respectively. Previous study has revealed these mutations could be nodule-specific [1, 22], which might be associated with multiple individual macronodule formations although the mechanisms in these mutation-mediated PBMAH need to be further studies.

For patients with no evidence of cortisol excess at diagnosis, active surveillance is proposed with annual clinical and biochemical assessment. On the other hand, for patients with overt CS or clinical consequences of hypercortisolism (i.e., diabetes, hypertension, and bone fragility), a surgical or medical treatment should be taken into consideration. Steroid synthesis inhibitors including metyrapone and ketoconazole [23], mifepristone and glucocorticoid receptor antagonist [24], and mitotane [25] were the suggested medical treatment. Bilateral total adrenalectomy has been the mainstay of the treatment for PBMAH with CS [26], but life-long corticosteroid replacement is needed and associated health care burden including risk of adrenal crisis are unavoidable. Unilateral adrenalectomy has less complications [27] although recurrence of hypercortisolism exists [19, 28]. Recent studies suggest that total adrenalectomy of the larger adrenal and subtotal adrenalectomy of the contralateral adrenal (adrenal-sparing surgery) could be the better choice [29]. However, it remains challenging to decide which adrenal should be removed first and to what extent the subtotal adrenalectomy should be conducted on the contralateral adrenal. Our AVS results confirmed that the right adrenal with larger sizes of nodules produced more cortisol and thus the right adrenalectomy was initially performed.

In conclusion, PBMAH patient with *ARMC5* mutations has severe CS symptoms and serious complications. Somatic *ARMC5* mutations might be nodule-specific. AVS combined with the sizes of the nodules is helpful in identifying the dominant side of bilateral adrenal lesion of cortisol secretion.

## Supplementary Information

**Additional file 1: Supplementary Table 1.** Identified other 20 somatic single nucleotide variants (SNVs)/insertion-deletion (indel) mutations in the right adrenal mass.

**Additional file 2: Supplementary Table 2.** Identified other 25 somatic single nucleotide variants (SNVs)/insertion-deletion (indel) mutations in the left adrenal mass.

**Additional file 3.** CARE Checklist of information to include when writing a case report.

## Data Availability

The raw sequencing data from this study have been deposited in the Genome Sequence Archive (GSA) with the project number of PRJCA012523.

## Abbreviations

ARMC5: Armadillo repeat containing 5
PBMAH: Primary bilateral macronodular adrenal hyperplasia
CS: Cushing syndrome
SCS: Subclinical Cushing syndrome
CT: Computed tomography
WES: Whole-exome sequencing
AVS: Adrenal venous sampling
LD-DST: Low-dosed dexamethasone suppression test
HD-DST: High-dosed dexamethasone suppression test
PPNAD: Primary pigmented nodular adrenal disease
AIMAH: ACTH-independent macronodular adrenal hyperplasia
