# Supplementary material for: Primary bilateral macronodular adrenocortical hyperplasia (PBMAH) patient with ARMC5 mutations
Source: BMC Endocr Disord. 2023 Apr 7;23:77. doi: 10.1186/s12902-023-01324-3 (PMC10080789; doi:10.1186/s12902-023-01324-3)
Supplement: Supplementary file 1 — Additional file 1: Supplementary Table 1. Identified other 20 somatic single nucleotide variants (SNVs)/insertion-deletion (indel) mutations in the right adrenal mass. [file 12902_2023_1324_MOESM1_ESM.docx]

Supplementary table 1. Identified other 20 somatic single nucleotide variants (SNVs)/insertion-deletion (indel) mutations in the right adrenal mass.

| Gene symbol | Transcripts | Genomic location | | | cHGVS | pHGVS | ExIn ID | Mutation frequency |
| --- | --- | --- | --- | --- | --- | --- | --- | --- |
| *NLN* | NM_020726.4 | | 5q12.3 | c.1988_1990delinsATG | | p.M663_K664delinsNE | EX13 | 19.2% |
| *ANKRD13C* | NM_030816.4 | | 1p31.1 | c.92C>T | | p.A31V | EX1 | 18.6% |
| *TMEM143* | NM_018273.2 | | 19q13.32 | c.230T>G | | p.F77C | EX2 | 18.1% |
| *EPHA5* | NM_004439.5 | | 4q13.1-q13.2 | c.2550delA | | p.I850Mfs*17 | EX15 | 11.9% |
| *RAD54L* | NM_001142548.1 | | 1p34.1 | c.409G>C | | p.E137Q | EX7 | 9.1% |
| *MRS2* | NM_020662.2 | | 6p22.3 | c.1205C>T | | p.A402V | EX10 | 8.4% |
| *DNAH5* | NM_001369.2 | | 5p15.2 | c.9443G>A | | p.C3148Y | EX56 | 7.3% |
| *PEX13* | NM_002618.3 | | 2p15 | c.416C>T | | p.A139V | EX2 | .o;6n.8% |
| *ITGB8* | NM_002214.2 | | 7p21.1 | c.673A>C | | p.I225L | EX5 | 6.1% |
| *ASPM* | NM_018136.4 | | 1q31.3 | c.5632G>A | | p.V1878M | EX18 | 5.9% |
| *GLUL* | NM_002065.5 | | 1q25.3 | c.776A>G | | p.K259R | EX7 | 5.6% |
| *UBXN1* | NM_015853.3 | | 11q12.3 | c.26G>A | | p.S9N | EX1 | 5.5% |
| *LOC100507656* | XM_003118753.2 | | - | c.1627G>A | | p.V543M | EX2 | 5.1% |
| *CLTC* | NM_004859.3 | | 17q23.1 | c.852_868delCTATGATCTTGAGACTG | | p.D286Lfs*6 | EX6 | 3.7% |
| *KLC1* | NM_001130107.1 | | 14q32.33 | c.1092delA | | p.A365Pfs*28 | EX8 | 3.3% |
| *HOXC11* | NM_014212.3 | | 12q13.13 | c.409T>G | | p.F137V | EX1 | 3.1% |
| *NUFIP1* | NM_012345.2 | | 13q14.12 | c.1036G>A | | p.E346K | EX8 | 2.4% |
| *OTOG* | XM_291816.8 | | 11p15.1 | c.7906C>T | | p.R2636C | EX47 | 2.2% |
| *TTN* | NM_001267550.1 | | 2q31.2 | c.56056C>A | | p.P18686T | EX289 | 2.1% |
| *TARBP1* | NM_005646.3 | | 1q42.2 | c.4184A>G | | p.Y1395C | EX26 | 2.0% |

HGVS, Human Genome Variation Society; ExIn, exon-intron.
