# Supplementary material for: Primary bilateral macronodular adrenocortical hyperplasia (PBMAH) patient with ARMC5 mutations
Source: BMC Endocr Disord. 2023 Apr 7;23:77. doi: 10.1186/s12902-023-01324-3 (PMC10080789; doi:10.1186/s12902-023-01324-3)
Supplement: Supplementary file 2 — Additional file 2: Supplementary Table 2. Identified other 25 somatic single nucleotide variants (SNVs)/insertion-deletion (indel) mutations in the left adrenal mass. [file 12902_2023_1324_MOESM2_ESM.docx]

Supplementary table 2. Identified other 25 somatic single nucleotide variants (SNVs)/insertion-deletion (indel) mutations in the left adrenal mass.

| Gene symbol | Transcripts | Genomic location | cHGVS | pHGVS | ExIn_ID | Mutation frequency |
| --- | --- | --- | --- | --- | --- | --- |
| *RGS3* | NM_144489.2 | 9q32 | c.245C>T | p.A82V | EX1 | 17.4% |
| *NCEH1* | NM_001146276.1 | 3q26.31 | c.19C>T | p.Q7* | EX1 | 17.2% |
| *RBP1* | NM_002899.3 | 3q23 | c.50C>T | p.A17V | EX1 | 14.8% |
| *SLC28A1* | NM_004213.3 | 15q25.3 | c.1763-2A>T | . | IVS17 | 13.6% |
| *FOXD3* | NM_012183.2 | 1p31.3 | c.64G>A | p.D22N | EX1 | 4.9% |
| *USP9X* | NM_001039590.2 | Xp11.4 | c.5251C>A | p.L1751I | EX34 | 4.9% |
| *USP48* | NM_032236.5 | 1p36.12 | c.2165A>G | p.N722S | EX17 | 4.5% |
| *NCBP1* | NM_002486.4 | 9q22.33 | c.322A>G | p.M108V | EX4 | 4.1% |
| *DSCAML1* | NM_020693.2 | 11q23.3 | c.118A>T | p.R40* | EX1 | 4.0% |
| *PHOX2B* | NM_003924.3 | 4p13 | c.741_758delCGCGGCAGCGGCGGCGGC | p.A241[20>14] | EX3 | 3.8% |
| *ATXN2L* | NM_148414.2 | 16p11.2 | c.251C>A | p.P84Q | EX1 | 3.5% |
| *DSP* | NM_004415.2 | 6p24.3 | c.2270A>G | p.N757S | EX16 | 3.3% |
| *DENND4B* | NM_014856.2 | 1q21.3 | c.16C[7>6] | p.R8Gfs*8 | EX2 | 3.2% |
| *LINGO3* | NM_001101391.1 | 19p13.3 | c.1057C>A | p.P353T | EX2 | 2.7% |
| *CXorf27* | NM_012274.1 | Xp11.4 | c.289A>G | p.S97G | EX1 | 2.7% |
| *FBXO41* | NM_001080410.2 | 2p13.2 | c.165_179delGGCCGCCGCCGCCGC | p.A51[11>6] | EX1 | 2.5% |
| *CCNI* | NM_006835.2 | 4q21.1 | c.463C>T | p.H155Y | EX6 | 2.5% |
| *ZNF205* | NM_003456.2 | 16p13.3 | c.1628C>G | p.A543G | EX7 | 2.5% |
| *TWISTNB* | NM_001002926.1 | 7p21.1 | c.32C>T | p.P11L | EX1 | 2.3% |
| *MYH1* | NM_005963.3 | 17p13.1 | c.3293T>C | p.I1098T | EX26 | 2.3% |
| *MBLAC1* | NM_203397.1 | 7q22.1 | c.253G[5>4] | p.G86Afs*22 | EX2 | 2.1% |
| *KIAA1324* | NM_020775.4 | 1p13.3 | c.1802C>T | p.S601F | EX14 | 2.0% |
| *SCN3A* | NM_006922.3 | 2q24.3 | c.3736G>T | p.V1246F | EX21 | 2.0% |
| *KIAA1147* | NM_001080392.1 | 7q34 | c.1269G>C | p.M423I | EX9 | 2.0% |
| *USP47* | NM_017944.3 | 11p15.3 | c.3309G>C | p.L1103F | EX23 | 2.0% |

HGVS, Human Genome Variation Society; ExIn, exon-intron.
